# eHealth literacy in emergency care: a scoping review

**DOI:** 10.1186/s12913-026-14637-5

**Published:** 2026-05-07

**Authors:** Julia Paller, Arvid Steinar Haugen, Franziska Großschädl

**Affiliations:** 1 Department of Nursing and Health Promotion – Acute and Critical Illness, Faculty of Health Sciences, Oslo Metropolitan University, P.O. Box 4, St. Olavs Plass, Oslo, 0130 Norway; 2 Department of Anaesthesia and Intensive Care, Haukeland University Hospital, Bergen, Norway; 3 Institute of Nursing Science, Medical University Graz, Graz, Austria

**Keywords:** eHealth literacy, Digital health literacy, Emergency care, Emergency Department (ED), Scoping review

## Abstract

**Background:**

With the rise of online health information (OHI), eHealth literacy has become increasingly important. The e-health literacy framework (eHLF) categorizes key skills necessary for effective eHealth literacy. Patients’ limited health literacy correlates with preventable, non-urgent emergency department (ED) visits, contributing to overcrowding, increased costs, and stress among healthcare professionals. However, the role of eHealth literacy in the ED remains unclear. Understanding its impact is essential to provide insight into overcrowding and internet use in the ED. This scoping review aimed to investigate eHealth literacy in emergency care.

**Methods:**

A scoping review was conducted following the guidelines of the Joanna Briggs Institute (JBI). An initial literature search was conducted in March 2024, followed by a systematic literature search in May 2024 using the databases PubMed, CINAHL, and Ovid, supplemented by Google Scholar, reference lists, and library sources. Inclusion and exclusion criteria were defined based on the PCC mnemonic. After study selection, the studies were critically appraised by two researchers using JBI checklists. Data was extracted and presented both narratively and in tabular form.

**Results:**

Nine studies were evaluated, and results were categorized as “individual”, “interaction”, and “system” based on the eHLF. The primary objectives were to examine internet use before ED visits and its correlations with anxiety and cyberchondria – excessive online searching leading to unfounded concerns about physical health. Findings showed widespread internet use for health-related information, though most did not use it to decide whether to visit the ED. Some studies found increased searching correlated with anxiety and cyberchondria. Other aspects included the effect on the doctor-patient relationship, trust, access and understandability of OHI.

**Conclusion:**

Internet use before ED visits is common and access to digital services is widespread. Some studies link increased internet searches to higher cyberchondria and impacts on the doctor-patient relationship. However, gaps remain in tailored digital devices and understanding eHealth literacy’s association with demographic factors. Both patients and healthcare providers in the ED require education on using OHI. Health professionals should acknowledge internet use before ED visits and discuss online findings with patients. Government guidelines and support programs should help users make informed health decisions.

**Supplementary Information:**

The online version contains supplementary material available at 10.1186/s12913-026-14637-5.

## Background

The Emergency Department (ED) is part of the healthcare system that focuses on treating critically ill or injured patients [1]. Trends show that the number of non-urgent patients visiting the ED is rising [2, 3], leading to overcrowding [4, 5]. Overcrowding in the ED is an international problem that can result in inadequate emergency care and reduced quality of care [1, 6]. The consequences range from longer waiting times for patients to more medical errors and higher mortality rates [5–7]. Furthermore, it can decrease health professionals’ job satisfaction and increase their stress level and risk of burnout [5, 8] and negatively impact institutional costs [5–7]. Reducing the number of non-urgent ED visits, and thereby reducing overcrowding, is essential for ensuring patient safety and supporting the healthcare system [5–7]. One reason for non-urgent ED visits is limited health literacy [9]. Individuals with limited health literacy are often unable to make informed decisions about their health and may struggle to apply health information appropriately to their own health problems [10]. A study demonstrates a correlation between non-urgent ED visits and health literacy, as patients with higher health literacy are 2.3 times less likely to visit the ED with a preventable condition [9].

The term “health literacy” has many different definitions that have changed over time. Nutbeam [10] proposed an influential definition that goes beyond viewing health literacy as the ability to read health information, by also incorporating empowerment, motivation, and interaction. Therefore, this article adopts Nutbeam’s [10] definition. Health literacy, in this context, combines basic skills like reading and writing with more advanced skills, such as being able to access health information, critically appraise it and apply it to manage one’s life [10]. High health literacy has been associated with positive health outcomes, including increased well-being and safety, as well as enabling individuals to make informed health-related decisions [11]. Conversely, low health literacy has been linked to knowledge gaps about diseases and conditions, reduced self-reported health, increased ED revisits and higher institutional costs [12, 13].

In 2024, approximately 67.1% of the global population used the internet, with this trend steadily rising [14]. In 2020, more than 50% of EU citizens searched for health-related information online [15]. These statistics highlight the importance of not only focusing on health literacy but also on digital health literacy, commonly known as eHealth literacy [16].

EHealth literacy is the ability to access, locate, understand, appraise and apply health information from online sources to manage a health problem [17]. Norgaard et al. [18] developed a model for an interpretation of eHealth literacy, known as the e-health literacy framework (eHLF), which integrates perspectives from various stakeholders, making it widely applicable. By incorporating both individual competencies and system-related factors, along with aspects of interacting with digital devices and online health information, the framework provides a comprehensive description of the concept of eHealth literacy. The eHLF is therefore a useful tool for categorizing evidence on a given topic and gaining an overview. The eHLF consists of three main domains: “individual,” “interaction,” and “system”. The domain “individual” includes individual competence, while the domain “system” encompasses access and the overall characteristics of digital services. The domains “individual” and “system” overlap to form the domain “interaction”, which incorporates characteristics of both individuals and the system. It refers to users’ engagement, feelings, and motivation when using the internet. These three main domains are further divided into seven subcategories: “ability to process information”, “engagement in own health”, “ability to actively engage with digital services”, “feeling safe and in control”, “motivated to engage with digital services”, “access to digital services that work”, and “digital services that suit individual needs” [18].

Access to online health information at any time and by any individual has both positive and negative effects. Studies have shown that the internet can serve as a valuable resource to help patients better understand their diagnoses and encourage them to ask questions during doctor visits [19]. Furthermore, it can increase self-empowerment in managing health conditions and improve overall quality of life [20]. However, searching for health-related information online also presents several challenges and potential negative consequences. These include reduced medication adherence and increased concerns about medications and treatment plans [21]. In addition, due to the vast amount of health information published online in a short period of time, the risk of encountering misinformation is significant. The spread of misleading content continues to rise, and several studies have identified misinformation on various health-related topics [22–24], which can lead individuals to make harmful decisions about their health [25, 26]. Moreover, excessive internet searching for health information can result in conditions such as cyberchondria, a phenomenon in which individuals experience unfounded health anxiety due to compulsive online searches [27].

The European Health Literacy Population Survey examined health literacy and eHealth literacy across 17 countries from 2019 to 2021 [28]. Results on eHealth literacy showed a mean score of 62.5 out of possible 100 points, with country-specific scores ranging from 41.8 in Germany to 78.7 in Norway [28]. Participants faced challenges in determining the reliability of online health information (OHI), recognizing whether the information had commercial bias and effectively applying it to manage their own health [28]. The survey underscores the need to improve eHealth literacy skills. Online health information should be accessible, easy to understand, trustworthy, user-friendly and applicable [28].

Despite evidence demonstrating the relevance of this topic, research on eHealth literacy in the ED remains limited. Research focusing on EDs suggests a correlation between health literacy with non-urgent emergency visits [9, 29] and patient outcomes [30]. Studies on eHealth literacy primarily focus on measurement tools [31, 32] or examine different populations [33, 34]. There are no recent reviews exploring the concept eHealth literacy in the ED. Given this, the aim of this review is to investigate how patients’ eHealth literacy applies in emergency care. The following research question will be explored: How does eHealth literacy apply in the context of emergency care?

## Methods

### Design

A scoping review was conducted, as this study design is suitable for topics with diverse and heterogeneous literature [35]. It allows for the inclusion of various study designs and enables mapping of the existing literature, providing a broad overview of the topic [36]. The scoping review followed the systematic approach outlined by the Joanna Briggs Institute (JBI) [35] and was further based on the Preferred Reporting Items for Systematic reviews and Meta-Analyses extension for Scoping Reviews (PRISMA-ScR) guidelines [37], which can be found in the supplementary files [see Additional file 1].

### Search strategy

First, a formal study plan was written prior to the start of the study, outlining key elements of how to conduct this work. The study plan was written by one author (JP) and reviewed by another (FG) at the Medical University of Graz, Austria. However, it was not registered in a database for systematic reviews. In March 2024, an initial data search was conducted, followed by a systematic literature search in May 2024. The databases *Public Medical Literature Online (PubMed)*, *Cumulative Index to Nursing and Allied Health Literature (CINAHL)* and *Embase and Cochrane Central Register of Controlled Trials (CENTRAL)* via Ovid were searched. In addition, the top 100 search results from Google Scholar along with selected library records from the Medical University of Graz were reviewed. Furthermore, references from the included studies were examined.

### Eligibility criteria

Inclusion and exclusion criteria were defined based on the PCC (Population, Concept, Context) mnemonic [35]. Searching for OHI is common among children and adolescents. However, searching focuses primarily on topics like sexual health, mental health or chronic conditions. They also use the internet for health-related networking and communication, while searches for severe diseases or acute injuries are limited [38]. Additionally, no research exists on the internet use of children or adolescents presenting to the ED. To understand eHealth literacy among the general ED population, this review focuses on adults, including only studies involving individuals aged 18 and older. To capture the full scope of this topic, addressing the concept of eHealth literacy were considered. Beyond the general concept of eHealth literacy [17], studies involving adults who interact with health information from electronic sources and the consequences of this internet use were also considered. To gain an overview of the topic, evidence from all points of the patient care journey were considered. This includes patients who searched for health-related information online before, during, or after their ED visit.

Regarding the context, only studies conducted in a general ED were included. Additionally, only studies published in English or German met the inclusion criteria. To provide a comprehensive overview of the topic, no time restrictions were applied, and all study designs were included. Table 1 summarizes the inclusion and exclusion criteria.

**Table 1:** Inclusion and exclusion criteria based on the PCC mnemonic [33]

	Inclusion criteria	Exclusion criteria
Population	adults presenting to the ED	children up to 18
Concept	eHealth literacy interaction with online health information consequences of the interaction	quality assessment of online health information/websites digital interventions
Context	general ED	psychiatric ED pediatric ED primary care

PCC = Population, Concept, Context; ED = emergency department

Search strategies were developed by two researchers (JP, FG) for each database, incorporating keywords based on the PCC mnemonic. Synonyms, along with Medical Subject Headings (MeSH Terms) in PubMed and Major Headings (MH) in CINAHL, were included. The keywords, synonyms, MeSH Terms and MH were combined into search strings using the Boolean operators “AND” and “OR”. Additionally, truncations (*) were applied. The search strings were discussed and adapted multiple times during scientific colloquium sessions at the Medical University of Graz, involving two authors of this article (JP, FG) and participants with different health professional backgrounds. The complete search strategies can be found in Additional file 2.

### Study selection

**Fig. 1:**
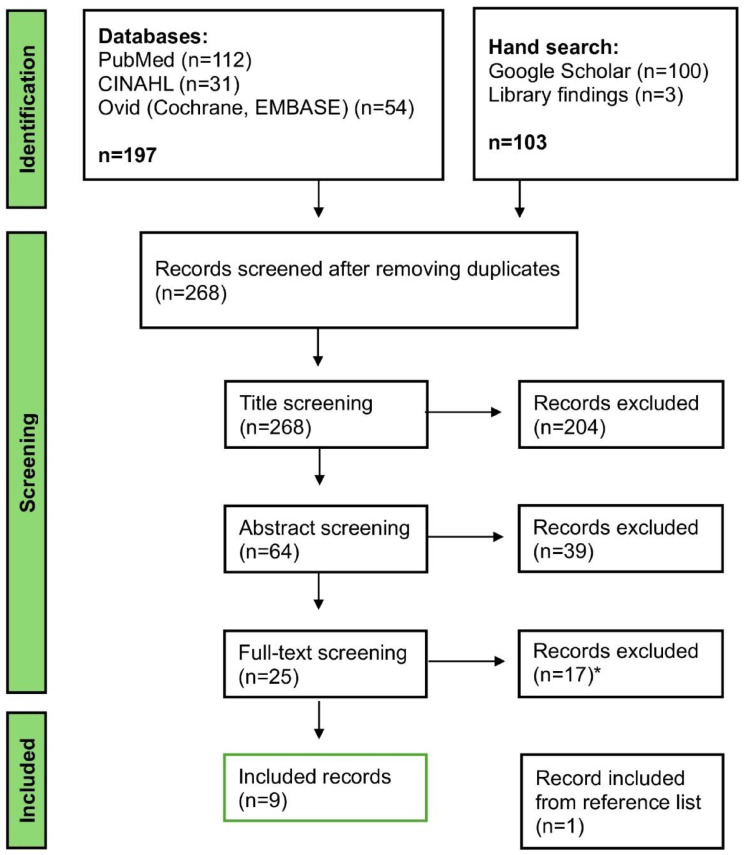
Flow chart demonstrating the screening process derived from Page et al. [36]. * Reasons for exclusion: setting/context not fitting (*n* = 9), evaluation of websites/interventions (*n* = 3), population not fitting (*n* = 1), general health literacy (*n* = 4)

Incorporating identified studies from databases (*n* = 197) and hand-searching (*n* = 103), a total of 300 eligible studies were identified and exported into *Endnote 20*. Afterwards, they were screened for eligibility based on predefined inclusion and exclusion criteria and duplicates were removed. The titles of the remaining 268 studies were screened, leaving 64 studies for abstract screening. After excluding 39 records, 25 were screened full text. 17 records were excluded, leaving eight studies, whose reference lists were examined, resulting in the inclusion of one additional study. After the screening process, nine eligible studies were identified. The screening process, based on Page et al. [39], is illustrated in Fig. 1. The study selection was carried out by one author (JP).

### Critical appraisal

The critical appraisal was conducted using quantitative and qualitative critical appraisal tools of the Joanna Briggs Institute (JBI) [40–42]. Depending on the study design, the checklists included eight to 13 questions with the answer options: “yes”, “no”, “unclear” and “not applicable”. The critical appraisal is presented narratively in the results section. However, to provide a comprehensive overview of the existing literature, methodological quality was not used as an exclusion criterion. The appraisal was conducted by two authors (JP, ASH) independently, and any inconsistencies were discussed until a consensus was reached. The complete critical appraisals are available in Additional file 3.

### Data extraction and synthesis

Data were extracted based on the JBI guidelines by two authors (JP, FG) [35]. A table of study characteristics, including the author(s) and year, location, objective(s), study design, sample size, outcomes and main results, was created. Demographic data and all results relevant to this study’s aim were extracted and summarized narratively. Furthermore, the results were categorized using the eHLF [18]. Table 2 illustrates the eHLF, including its domains, subcategories, and descriptions.

**Table 2:** Domains, subcategories, and descriptions of the eHLF [18]

Domain	Subcategory	Description
individual	Ability to process information	Ability to read, understand, find and apply information
	Engagement in own health	Managing one’s own health and navigating the healthcare system to support individual needs
interaction	Ability to actively engage with digital services	Capacity to access systems and effectively use technology and digital devices
	Feel safe and in control	Trust in the information found, as well as in technology and digital devices; data security
	Motivated to engage with digital services	Motivation and interest in using technology
system	Access to digital services that work	Ability to access technology, systems, software and hardware; systems that function properly are user-friendly and can be effectively used by individuals
	Digital services that suit individual needs	Systems that are tailored to the user’s specific needs

## Results

Nine studies were included, of which six were carried out in the United States (U.S.) [43–48]. The remaining studies were conducted in Australia [49], Pakistan [50] and Canada [51]. All studies involved patients visiting the ED and aimed to determine general internet searching characteristics [43, 47, 49] as well as search accuracy [45, 50]. Other objectives included investigating the impact of internet searching on the decision to seek care in the ED [46, 48] and its effect on cyberchondria and anxiety [50, 51]. Sample sizes ranged from 74 [48] to 723 [47] participants. The study characteristics are presented in Table 3. First, the quality of the included studies is described. Furthermore, the results are presented based on the domains and subcategories derived from the eHLF [18]. Due to the lack of evidence, the chapters vary in length—reporting more results in some domains while only briefly addressing others.

**Table 3:** Study characteristics of the included studies (*n* = 9)

Author(s), year	Country	Objective(s)	Study design	Sample size (*n*)	Main outcome(s)
Asch et al., 2019	United States	Testing patients’ willingness to share Google search histories with their EMR^1^ and exploring the association between internet searches and clinical presentation	Cross-sectional	*n* = 334 (willing to share EMR^1^) *n* = 103 (final sample)	o EMR data o Google searches
Cocco et al., 2018	Australia	Determination of prevalence, predictors and characteristics of internet searches, effect on doctor-patient relationship and treatment compliance	Multi-center, observational cross-sectional	*n* = 400	o Prevalence, characteristics o Doctor-patient relationship o Treatment compliance o Predictors of searching o EHealth literacy
Malik et al., 2019	Pakistan	Assessment of cyberchondria	Cross-sectional	*n* = 304	o Prevalence of cyberchondria
Martin et al., 2019	United States	Determining the accuracy of online searching and evaluating the impact on anxiety	RCT	*n* = 300 No Search: *n* = 101 Google Search: *n* = 100 HFD: *n* = 99	o Accuracy of patient generated differential by matching clinicians’ differential (at least 2 out of 3) o Anxiety
McCarthy et al., 2017	United States	Characterizing internet searches evaluating the relationship to clinical diagnoses	Cross-sectional with qualitative analyses	*n* = 170	o Search terms o Relationship between search term and final diagnosis
Pourmand & Sikka, 2011	United States	Impact of online searching on decision to seek the ED	Cross-sectional	*n* = 489	o Prevalence of online searching o Impact to seek the ED
Rutty, 2023	Canada	Evaluating attitude towards OHI, prevalence of cyberchondria and health anxiety	Mixed-methods	Quantitative *n* = 128 Qualitative *n* = 101 (*n* = 9 interviews)	o Characteristics of internet use o Anxiety o Cyberchondria o EHealth literacy o Experiences
Scott et al., 2015	United States	Analyzing OHI utilization across age groups	Cross-sectional	*n* = 723	o Access o Understanding o Trust o Characteristics of internet use o Impact to seek the ED
Yastik, 2017	United States	Describing OHI seeking and the impact on decision to visit ED by non-urgent patients	Descriptive/correlation	*n* = 74	o Characteristics of internet use o Impact to seek the ED

Abbreviations: EMR = Electronic Medical Record; RCT = Randomized-controlled trial; HFD = Google Search with health-related features disabled; OHI = Online health information

### Quality of the included studies

The quality of all included studies was assessed through critical appraisal using JBI checklists. Seven cross-sectional studies [43, 45–50], one randomized-controlled trial [44] and one mixed-methods study [51] were included in this scoping review. Overall, the cross-sectional studies used clear methods to measure the exposure and the condition objectively, reliably and validly. Nevertheless, confounding factors were poorly described. Only one study focused on age groups and stratified results. Moreover, it had a large sample size, with 723 participants [47]. Conversely, Malik et al. [50] demonstrated limited study quality with missing information about inclusion criteria, study subjects and setting. The mixed-methods study met nearly all requirements for a methodologically high-quality study, with only a few missing details about the researcher [51]. Similarly, the randomized-controlled trial by Martin et al. [44] demonstrated overall good quality. However, blinding was not feasible, as the intervention was self-administered. Participants were divided into three groups: no searching, Google searching with a symptom search tool, and Google searching with disabled health features. The aim was to assess the accuracy of self-reported diagnoses compared to clinical diagnoses. Another unclear aspect was whether all outcome measurements were reliable [44].

### Individual

Based on the eHLF, the first domain is called “individual” and is further divided into two subcategories. The first subcategory, “the ability to process information”, encompasses the ability to read, write and cope with health-related information found on the internet [18]. Four studies examined the effect of internet searching on anxiety [44, 49–51]. One of these studies [51] examined not only anxiety but also cyberchondria, including possible correlations between the two. Another study [50] also addressed the topic of cyberchondria; however, it did not report results on the overall Cyberchondria Severity Scale (CSS) score. Instead, it only described specific questions from the CSS.

Results showed that 39.9% of participants in the study by Cocco et al. [49] became anxious or worried while searching for OHI. Another study found that anxiety levels were significantly lower when participants either did not search online or used Google with built-in health-related features that directed users to physician-approved information [44]. Malik et al. [50] reported that about 70% of participants often or always felt anxious after researching symptoms online. More than two-thirds panicked when information on the internet suggested their condition is rare or serious and nearly one-third experienced trouble sleeping after online searches [50]. In the study by Rutty [51], 13.3% of participants had high levels of anxiety, as measured by the Short Health Anxiety Inventory (SHAI). The SHAI score ranges from 0 to 42, with higher scores indicating greater health anxiety. A score of 20 is considered the threshold for a higher degree of anxiety. Furthermore, the study identified a correlation between age and anxiety, with younger participants being more anxious than those over 50 (*p* = 0.008, 95% CI [1.10,9.99]) and those over 65 (*p* < 0.001, 95% CI [2.30–11.4]). Moreover, participants aged 50 to 64 reported higher anxiety levels than those over 65. Participants who searched for information before visiting the ED exhibited significantly higher anxiety levels (*p* = 0.004).

Cyberchondria refers to excessive, frequent online searching and exposure to vast amounts of health information, leading to increased anxiety levels [27]. Rutty [51] found that nearly one third of the participants demonstrated increased levels of cyberchondria. Various significant correlations were found, including a link between consulting a health professional due to OHI and higher cyberchondria and anxiety scores. Moreover, eHealth literacy was positively correlated with cyberchondria, but no association was found between eHealth literacy and health anxiety. Qualitative findings also suggested a correlation between searching for OHI and increased anxiety and cyberchondria, as interviewed participants reported feeling overwhelmed and panicked while searching online [51].

The second subcategory, “engagement in own health” refers to the ability to manage personal health, take responsibility and navigate the healthcare system [18]. Most studies found that most participants did not use the internet to decide whether to visit the ED [46–48]. Additionally, 17% [46] or 43.2% [48] ultimately changed their decision to visit the ED based on online searches. However, Rutty [51] reported that over 50% of participants received recommendations from online sources to visit the ED, and about half of them followed these recommendations the same day. Interviews also revealed that participants viewed the internet as a potential first step in engaging with the healthcare system [51].

### Interaction

The first subcategory in the domain “interaction” is called “ability to actively engage with digital services” and includes the readiness to use electronic devices and digital services [18]. Therefore, all results regarding the characteristics of internet usage were included in this subcategory.

Nearly all studies have explored general internet usage characteristics. The prevalence of internet searching for health-related information ranged from 37.2% [48] to 49% (95% CI, 44.1%-53.9%) [49], and to 73% [48]. Rutty [51] found that 93.8% of participants searched for OHI in the past year. Among them, 35.9% searched a few times a year, 39.8% occasionally checked their symptoms online and 45.3% primarily use the internet for health-related information.

Younger participants were more likely to use the internet for health-related information [47–49]. Between 15% and 63.3% of participants searched for OHI before presenting to the ED [43–45, 51]. Nearly one-third searched for their symptoms online more than 24 h after first experiencing them [48], while another study reported that nearly two-thirds searched when they felt an unexplained bodily sensation [50]. Participants primarily searched for chief complaints [43, 49] and symptoms [45, 49] followed by treatment options [49], diagnoses [45] and ED-related information, such as directions [43].

Furthermore, participants with a higher level of education were nearly twice as likely to search for health-related information online [48]. In contrast, Rutty [51] did not find a significant correlation between education and eHealth literacy. Additionally, no correlations were found between education and anxiety levels or cyberchondria [51]. Similarly, Cocco et al. [49] did not report any statistically significant correlations related to education.

On average, participants searched for 20 min, with search time increasing as the ED visit approached [49]. The median search time in the waiting room was 3.82 min [44]. Rutty [51] found that more than half of the participants searched for one to five hours, with longer search times significantly increasing anxiety and cyberchondria (*p* < 0.001). Two studies found that more than half of participants began their search using a search engine [48, 51]. The most commonly used search engine was Google [44, 49], although only 10.8% of participants in Yastik’s study [48] used Google. Other websites used included WebMD (24.3%), Yahoo (2.7%), Wikipedia (2.7%), and others. Half of the participants did not list any specific websites [48]. Other studies also reported frequent use of WebMD [45, 48], followed by hospital websites [45, 49] and online encyclopedias [49]. Hospital and university websites were the most trusted sources and smartphones were the most used devices [49].

Participants searched online for health-related information for various reasons, including curiosity about health conditions and a desire to better understand them. Additionally, OHI was seen as useful in conversations with health professionals, providing a sense of control and active engagement. Efficiency, speed and ease of internet use were also motivating factors. Due to a lack of trust in the healthcare system, some participants used the internet as a first step to assess their symptoms [51].

Another aspect investigated was the alignment of search terms with final diagnoses given by health professionals in the ED. A randomized-controlled trial demonstrated that online searching for health-related information had no effect on diagnostic accuracy [44]. McCarthy et al. [45] found that only 13% of participants who searched for a diagnosis received the same diagnosis in the ED. However, about two-thirds of participants showed near or complete concordance between their chief complaint and the final diagnosis.

The next subcategory “feel safe and in control” includes perceptions of and trust in technology and its safety [18]. Results showed that participants trusted health professionals more than the internet [47, 49–51]. Only 3% never trusted a health professional over the internet [50]. Another study reported that about one-third of participants had complete trust in health professionals [51]. Furthermore, information from health professionals was considered more trustworthy and understandable than online health information [47]. Additionally, 91% of participants stated they would never change their treatment plan based on differing information found on the internet [49].

The last subcategory in this domain is called “motivated to engage with digital services”. It includes people’s motivation to use the internet for health-related questions and highlights the benefits of internet use [18]. Results regarding participants’ motivation to actively discuss search results with health professionals and the general effect of searching on the doctor-patient relationship were included in this subcategory. More than three-fourths of the participants stated that searching for OHI had a positive influence on their relationship with their doctor, indicating that it helped them communicate, ask more informed questions and better understand medical information. About half of the participants felt that online searching empowered them and 42% agreed or strongly agreed that searching beforehand gave them more attention during the visit and enabled them to receive more information. An increase in the positive impact on the doctor-patient relationship also correlated with a higher eHEALS (e-health literacy score) [49]. However, Martin et al. [44] found no significant impact on the doctor-patient relationship. Two different studies demonstrated that about half of the participants shared information they found online with a health professional [48, 50].

### System

**Fig. 2:**
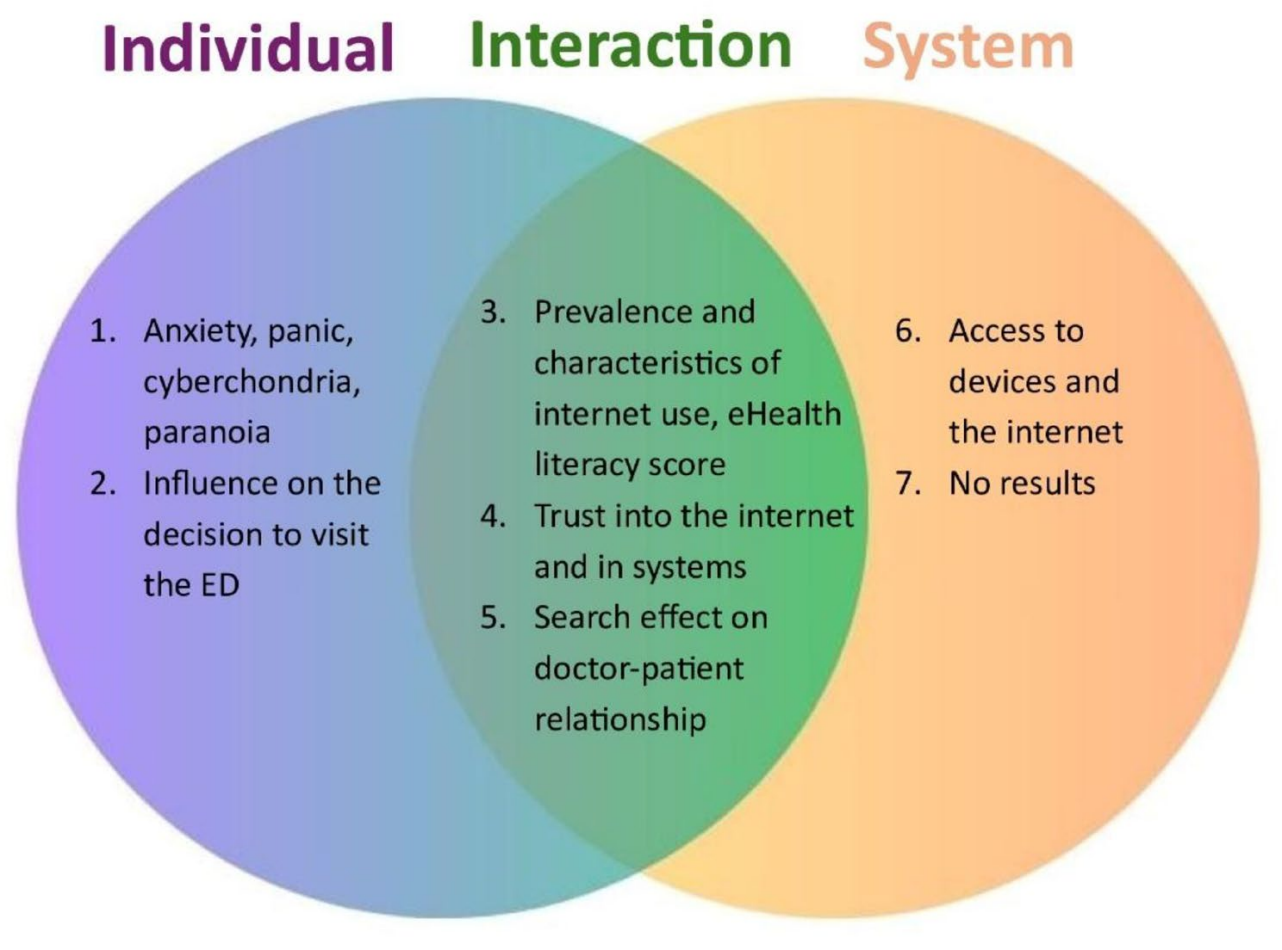
Illustration of the eHLF [18] including its three main domains and seven subcategories

The last domain “system” includes access to technology as well as systems, hardware and software, along with user-friendly interfaces. The first subcategory in this domain is called “access to digital services that work” and involves access to devices and understandable, easy-to-use hardware and software, including support systems [18]. Nearly all participants owned devices to search for OHI [44]. Other studies found similar results, indicating widespread access to electronic devices and the internet [46, 47]. However, internet access was significantly more prevalent among younger age groups, with 98% of participants aged 18 to 30 reporting access, whereas nearly three-quarters of individuals aged 75 and older reported having internet access (*p* < 0.001) [47]. Younger participants also perceived OHI as more accessible than information provided by healthcare professionals. Nevertheless, all age groups found information from physicians to be significantly more understandable (*p* = 0.511) and trustworthy (*p* = 0.061) [47]. Another study indicated that about half of the participants perceived OHI as easy to locate and understand [46]. The last subcategory “digital services that suit individual needs” refers to interfaces designed to accommodate individual needs, including user-friendly adaptations such as different language options and accessibility features for people with disabilities [18]. To this date, no research fitting this subcategory has been identified. Figure 2 illustrates the results categorized based on the eHLF [18] and Table 4 demonstrates the main findings of this scoping review in each domain.

**Table 4:** Main topics discussed in the included studies, categorized into the sub-categories of the eHLF [18]

Subcategories based on the eHLF [18]	Main topics discussed in the included studies
1. Ability to process information	Anxiety, panic, cyberchondria, paranoia
2. Engagement in own health	Influence on the decision to visit the ED
3. Ability to actively engage with digital services	Prevalence and characteristics of internet use, eHealth literacy score
4. Feel safe and in control	Trust into the internet and in systems
5. Motivated to engage with digital services	Search effect on doctor-patient relationship
6. Access to digital services that work	Access to devices and internet
7. Digital services that suit individual needs	No results

## Discussion

To address the research question, *“How does eHealth literacy apply in the context of emergency care?”* results from the included studies were categorized based on the eHLF [18]. Most studies fell into the category of “interaction”, focusing on internet use and showing varying prevalence in health-related searches [41–49]. Research on cyberchondria, anxiety, and panic shows mixed results. One study found mild associations [49], while others suggested that increased searching led to higher anxiety, panic, and cyberchondria [44, 50, 51]. One study demonstrated that internet searches empower patients in doctor-patient discussions [49], while Martin et al. [44] found no significant impact. In two studies, half of the participants shared online health information (OHI) with their doctors. Trust in online searches was limited, as patients were more likely to trust their physicians than internet search result [47, 49–51].

The study populations were predominantly young [44–46, 48, 49] and only one study stratified the population regarding age and oversampled older participants to gain generalizable data [47]. Younger participants were more likely to search for OHI and found it more accessible than visiting a general practitioner [47, 49, 51]. These results align with a cross-sectional study examining Egyptian adults, which also found that internet searching was more prevalent in younger participants [52]. Patients in the ED are on average over 75 years old [53, 54]. Therefore, the results of the included studies have to be interpreted cautiously since the lack of age stratification weakens their generalizability. In spite of that, internet use of the older generation is rising [55] and individuals over 75 represent the second-largest group presenting to the ED [56], with adults older than 50 being accountable for 40 million ED visits annually [57]. Since most ED patients are older [56–58] and internet use among this generation is increasing [55, 59], research about eHealth literacy focused on age is essential for obtaining representative data.

Furthermore, correlations between education and general health literacy have been found, suggesting that higher education is associated with greater health literacy [60, 61]. Moreover, higher education levels also enhance eHealth literacy skills [62]. Participants in the studies included in this scoping review generally have higher education levels, indicating a likelihood of greater eHealth literacy skills [44–49, 51]. Nevertheless, one study did not find a significant correlation between education and eHealth literacy [51]. This may be because the study sample predominantly consisted of individuals with higher education levels.

Another important factor that was not a focus of the included studies is language, as approximately 26 million people in the U.S. over the age of five have limited English proficiency [63]. Language barriers were not addressed because six studies included only participants who could understand and complete English surveys [44–49], one study included patients with basic skills in English or French [51] and two studies did not specify language requirements [43, 50]. This weakens the generalizability of the results, as language barriers hinder communication in emergency care and lead to increased resource use [64]. Patients who struggle to understand treatment plans may return to the ED [65]. Language proficiency is also correlated with health literacy, as individuals with high language proficiency tend to have higher health literacy [66]. Conversely, low language proficiency can lead to poorer health outcomes [67, 68], lower self-rated health [67] and difficulties understanding health-related information [69].

Additionally, the prevalence of internet searching for health-related information included studies that showed a wide range of the use of OHI, from 37.2% [46] to 93.8% [51]. Research indicates that about one in two EU citizens sought OHI in 2022 [70]. From July to December of the same year, 58.8% of U.S. citizens searched for OHI [71]. The low prevalence (37.2%) reported in the study by Pourmand and Sikka [46] could be explained by the early year, 2011, in which it was conducted, as internet use in the EU has increased significantly over the past 15 years [72]. The study by Rutty [51], conducted in 2023, supports this trend, as its reported prevalence is more than twice as high.

### Strengths and limitations

The key strength of this scoping review is its methodology, which employs a structured approach. The review is based on a clear research question and aim. A systematic search strategy was used, incorporating individually tailored search strings for different databases, supplemented by hand-searching. Additionally, no time limit was set, ensuring the inclusion of all relevant studies. The included studies were critically appraised using JBI checklists. The critical appraisal was conducted by two independent authors to reduce the risk of bias, and any discrepancies were discussed until a consensus was reached. Furthermore, the scoping review followed PRISMA-ScR guidelines. Another strength is the chosen study design. By conducting a scoping review, all relevant publications – both quantitative and qualitative – were included. To identify potential gaps, summarize findings and present the heterogeneous results, they were categorized based on the eHLF [18].

Nonetheless, only studies published in English or German were included and only three databases were searched. Furthermore, no librarian was involved in developing the search strategy. This may have led to the exclusion of potentially relevant studies. However, the search strings were discussed in detail and refined during colloquium sessions at the Medical University of Graz, with participants from various health professions. Nearly all studies were conducted in the U.S [43–48]., making it difficult to generalize the results due to international differences in healthcare systems [73]. While the study was not pre-registered and did not follow a priori protocol, it adhered to a structured study plan developed at the Medical University of Graz. Furthermore, study selection and data extraction were not blinded, as they were performed by a single author. Data synthesis and extraction did not follow a formal synthesis framework. However, the process closely adhered to the JBI guidelines and utilized the eHLF, a framework that describes the concept of eHealth literacy. Lastly, since the included studies are highly heterogeneous, the results could not be effectively compared.

### Implications for research and practice

Knowledge of eHealth literacy in the ED is essential, given the global issue of overcrowding [1]. Overcrowding negatively impacts patient care, healthcare professionals and institutional costs, affecting the entire emergency care system. Health literacy is associated with preventable ED visits [9, 13]. With the increasing role of technology in healthcare [15, 74], understanding patients’ internet behavior is crucial. The following research and practice implications, based on this review’s findings, aim to support patients, healthcare professionals and policymakers in addressing challenges related to OHI use and improving ED management.

The results of this scoping review highlight a research gap regarding the eHLF domain “system”, as only a few studies have addressed this topic. However, research about this topic is essential, given the increasing use of technology for health-related information. Evidence is particularly lacking in the subcategory of “digital services that suit individual needs.” Further research is needed to develop interfaces tailored to diverse users, such as incorporating multiple language options. Interfaces should also be adaptable for individuals with disabilities, for example, by offering settings that support blind users through text-to-speech functionality.

Furthermore, demographic factors influencing internet searches before an ED visit require further examination. Stratifying the population by age groups or educational levels - or focusing on specific groups - can enhance knowledge in this field. Since the ED population primarily consists of older adults [56, 57], and internet use in this group is increasing [55], age-specific research can improve understanding of the digital health behaviors of ED patients and support overall ED management. Additionally, multilingual surveys and interpreters can help overcome language barriers for a more representative sample.

To achieve a comprehensive understanding of the topic, various study designs must be conducted. On one hand, randomized controlled trials are needed to explore cause-and-effect relationships, for example, investigating correlations between eHealth literacy and cyberchondria. In interventions where participants are randomly assigned to a group that used the internet before visiting the ED and a group that did not, associations with demographic factors should be studied. On the other hand, qualitative studies can provide deeper insight into people’s experiences, emotions, needs, and challenges while searching online.

In practice, healthcare professionals must recognize the importance of internet searching before the ED visit. Therefore, this topic should be incorporated into education and training programs for healthcare professionals. Such training can help them better understand their patients and effectively address concerns arising from online searches. Patients should be supported through open discussions about online findings and encouraged to participate in shared decision-making. Additionally, healthcare professionals and hospitals should recommend high-quality medical websites and provide guidelines for safely and accurately searching for health-related information.

The increasing use of technology in healthcare introduces new demands, such as data protection and security [75, 76]. Training programs for healthcare professionals focused on technology and eHealth are therefore essential. Another practical implication relates to government regulations. Medical websites must adhere to guidelines and standards to ensure high-quality information.

Additionally, public involvement is crucial. Support should begin not only when patients present to the ED but also before they require acute care. Education on the use of digital devices and medical websites should be provided, with a particular focus on groups such as older adults, migrants and people with disabilities. Health policymakers must implement guidelines to promote user-friendly, accessible health websites while restricting the spread of misinformation. Furthermore, easily understandable, and accessible checklists can help patients navigate the internet to find reliable information and apply it effectively.

## Conclusion

The use of technology and the internet for online health information is increasing, but evidence on online health information in emergency departments remains limited. This scoping review explores the impact of eHealth literacy in emergency care. Studies covered various topics, including internet use prevalence, its effects, motivation, trust and understandability. However, no research on personalized digital services was identified.

Some findings suggested correlations between online health information searches and cyberchondria, anxiety and panic, while others show minimal association. Reported internet use for online health information varied widely. Most participants relied on Google as their primary search engine but did not use the internet to decide whether to visit the emergency department, trusting healthcare professionals more. Despite this, access to digital devices and online health information was widespread.

Heterogeneous study results limit comparability, highlighting the need for further research, particularly on demographic and language factors. Healthcare professionals must acknowledge patients’ internet use before the emergency department visit and guide them accordingly. Both patients and health professionals should be educated on effectively handling online health information. Governments should fund research regarding eHealth literacy in emergency departments and provide high-quality, accessible and easy-to-understand medical websites.

## Supplementary Information

Below is the link to the electronic supplementary material.

Supplementary Material 1: PRISMA-ScR checklist. Preferred Reporting Items for Systematic reviews and Meta-Analyses extension for Scoping Reviews (PRISMA-ScR) Checklist

Supplementary Material 2: Search strategies. Search strategies for each database developed by two authors (JP, FG)

Supplemetary Material 3: Critical appraisal. Description: Critical appraisals, based on the JBI checklists for analytical cross sectional studies [40], qualitative research [41] and randomized controlled trials [42] carried out by two independent authors (JP, ASH)

## Data Availability

The datasets used and/or analysed during the current study are available from the corresponding author on reasonable request.

## Abbreviations

CENTRAL: Cochrane Central Register of Controlled Trials
CINAHL: Cumulative Index to Nursing and Allied Health Literature
ED: Emergency Department
eHEALS: e-health literacy score
eHLF: e-health literacy framework
JBI: Joanna Briggs Institute
MeSH: Medical Subject Headings
MH: Major Headings
OHI: Online health information
PRISMA-ScR: Preferred Reporting Items for Systematic reviews and Meta-Analyses extension for Scoping Reviews
PubMed: Public Medical Literature Online
PCC mnemonic: Population, Concept and Context
