# Supplementary material for: eHealth literacy in emergency care: a scoping review
Source: BMC Health Serv Res. 2026 May 7;26:984. doi: 10.1186/s12913-026-14637-5 (PMC13371012; doi:10.1186/s12913-026-14637-5)
Supplement: Supplementary file 2 — Supplementary Material 2: Search strategies. Search strategies for each database developed by two authors (JP, FG) [file 12913_2026_14637_MOESM2_ESM.docx]

## **Search strings**

PubMed

112 results

((("electronic health literacy"[Title/Abstract] OR "mhealth literacy"[Title/Abstract] OR "online health literacy"[Title/Abstract] OR "digital health literacy"[Title/Abstract] OR "electronic health information"[Title/Abstract] OR "online health information"[Title/Abstract] OR "internet usage"[Title/Abstract] OR "digital health information"[Title/Abstract] OR "ehealth literacy"[Title/Abstract]) AND (emergency department* OR emergency room* OR emergency unit* OR "emergency care" OR "ED" OR "ER" OR emergency service, hospital[MeSH Terms] OR (emergency room visits[MeSH Terms])) AND (adult[MeSH Terms] OR adult OR aged OR middle aged OR young adult)))

CINAHL

31 results

S1 TI "electronic health literacy" OR TI "mhealth literacy" OR TI "online health literacy" OR TI "digital health literacy" OR TI "electronic health information" OR TI "online health information" OR TI "digital health information" OR TI "ehealth literacy"

S2 AB "electronic health literacy" OR AB "mhealth literacy" OR AB "online health literacy" OR AB "digital health literacy" OR AB "electronic health information" OR AB "online health information" OR AB "digital health information" OR AB "ehealth literacy" OR AB

S3 S1 OR S2

S4 emergency department* OR emergency room* OR emergency unit* OR "emergency care" OR "ED" OR "ER" OR MH emergency service, hospital OR MH emergency treatment OR “emergency treatment” OR MH emergency care

S5 adult OR MH adult OR aged OR middle aged OR young adult

S6 S3 AND S4 AND S5

Ovid (Embase, Cochrane)

54 results

EBM Reviews - Cochrane Database of Systematic Reviews <2005 to April 22, 2024>

Embase <1974 to 2024 April 30>

1 ("electronic health literacy" or "mhealth literacy" or "online health literacy" or "digital health literacy" or "electronic health information" or "online health information" or "digital health information" or "ehealth literacy" or “internet usage”).ti.

2 ("electronic health literacy" or "mhealth literacy" or "online health literacy" or "digital health literacy" or "electronic health information" or "online health information" or "digital health information" or "ehealth literacy" or “internet usage”).ab.

3 1 or 2

4 (emergency department* or emergency room* or emergency unit* or "emergency care" or "ED" or "ER").af.

5 (adult or aged or middle aged or young adult).af.

6 3 and 4 and 5

Google Scholar

100 results

(electronic health literacy OR online health literacy OR digital health literacy OR online health information OR ehealth literacy) AND (emergency department* OR emergency room* OR emergency unit* OR emergency care) AND (adult)
