# Supplementary material for: eHealth literacy in emergency care: a scoping review
Source: BMC Health Serv Res. 2026 May 7;26:984. doi: 10.1186/s12913-026-14637-5 (PMC13371012; doi:10.1186/s12913-026-14637-5)
Supplement: Supplementary file 3 — Supplemetary Material 3: Critical appraisal. Description: Critical appraisals, based on the JBI checklists for analytical cross sectional studies [40], qualitative research [41] and randomized controlled trials [42] carried out by two independent authors (JP, ASH) [file 12913_2026_14637_MOESM3_ESM.docx]

**Critical Appraisal**

**Checklist for cross sectional studies**

|  |  | Ash et al. (2019) | Cocco et al. (2018) | Malik et al. (2019) | McCarthy et al. (2017) | Pourmand & Sikka (2011) | Rutty (2023) | Scott et al. (2017) | Yastik (2017) |
| --- | --- | --- | --- | --- | --- | --- | --- | --- | --- |
| Question 1 | **JP**  **ASH**  **FINAL** | **Yes**  **Yes**  **Yes** | **Yes**  **Yes**  **Yes** | **No**  **No**  **No** | **Yes**  **Yes**  **Yes** | **Yes**  **Yes**  **Yes** | **Yes**  **Yes**  **Yes** | **Yes**  **Yes**  **Yes** | **Yes**  **Yes**  **Yes** |
| Question 2 | **JP**  **ASH**  **FINAL** | **Yes**  **Yes**  **Yes** | **Yes**  **Yes**  **Yes** | **No**  **No**  **No** | **Yes**  **Yes**  **Yes** | **Yes**  **Yes**  **Yes** | **Yes**  **Yes**  **Yes** | **Yes**  **Yes**  **Yes** | **Yes**  **Yes**  **Yes** |
| Question 3 | **JP**  **ASH**  **FINAL** | **N/A**  **Yes**  **Yes** | **N/A**  **Yes**  **Yes** | **N/A**  **Yes**  **Yes** | **Yes**  **Yes**  **Yes** | **N/A**  **No**  **No** | **N/A**  **Yes**  **Yes** | **N/A**  **Unclear**  **Unclear** | **N/A**  **Yes**  **Yes** |
| Question 4 | **JP**  **ASH**  **FINAL** | **N/A**  **Yes**  **Yes** | **N/A**  **Yes**  **Yes** | **N/A**  **Yes**  **Yes** | **Unclear**  **Unclear**  **Unclear** | **N/A**  **Yes**  **Yes** | **Yes**  **Yes**  **Yes** | **Yes**  **Yes**  **Yes** | **Yes**  **Yes**  **Yes** |
| Question 5 | **JP**  **ASH**  **FINAL** | **No**  **No**  **No** | **Yes**  **Yes**  **Yes** | **No**  **No**  **No** | **No**  **No**  **No** | **No**  **No**  **No** | **Yes**  **Yes**  **Yes** | **Yes**  **Yes**  **Yes** | **Yes**  **Yes**  **Yes** |
| Question 6 | **JP**  **ASH**  **FINAL** | **No**  **No**  **No** | **Yes**  **Yes**  **Yes** | **No**  **No**  **No** | **No**  **No**  **No** | **No**  **No**  **No** | **Yes**  **Yes**  **Yes** | **Yes**  **Yes**  **Yes** | **Yes**  **Yes**  **Yes** |
| Question 7 | **JP**  **ASH**  **FINAL** | **Yes**  **Yes**  **Yes** | **Yes**  **Yes**  **Yes** | **Yes**  **Yes**  **Yes** | **Yes**  **Yes**  **Yes** | **Yes**  **Yes**  **Yes** | **Yes**  **Yes**  **Yes** | **Yes**  **Yes**  **Yes** | **No**  **No**  **No** |
| Question 8 | **JP**  **ASH**  **FINAL** | **Yes**  **Yes**  **Yes** | **Yes**  **Yes**  **Yes** | **Unclear**  **Yes**  **Yes** | **Yes**  **Yes**  **Yes** | **Unclear**  **Yes**  **Yes** | **Yes**  **Yes**  **Yes** | **Yes**  **Yes**  **Yes** | **Yes**  **Yes**  **Yes** |

**Checklist for qualitative studies**

|  |  | Rutty (2023) |
| --- | --- | --- |
| Question 1 | **JP**  **ASH**  **FINAL** | **Yes**  **Yes**  **Yes** |
| Question 2 | **JP**  **ASH**  **FINAL** | **Yes**  **Yes**  **Yes** |
| Question 3 | **JP**  **ASH**  **FINAL** | **Yes**  **Yes**  **Yes** |
| Question 4 | **JP**  **ASH**  **FINAL** | **Yes**  **Yes**  **Yes** |
| Question 5 | **JP**  **ASH**  **FINAL** | **Yes**  **Yes**  **Yes** |
| Question 6 | **JP**  **ASH**  **FINAL** | **Yes**  **Unclear**  **Unclear** |
| Question 7 | **JP**  **ASH**  **FINAL** | **Yes**  **Unclear**  **Unclear** |
| Question 8 | **JP**  **ASH**  **FINAL** | **Yes**  **Yes**  **Yes** |
| Question 9 | **JP**  **ASH**  **FINAL** | **Yes**  **Yes**  **Yes** |
| Question 10 | **JP**  **ASH**  **FINAL** | **Yes**  **Yes**  **Yes** |

**Checklist for randomized controlled trials**

|  |  | Martin et al. (2019) |
| --- | --- | --- |
| Question 1 | **JP**  **ASH**  **FINAL** | **Yes**  **Yes**  **Yes** |
| Question 2 | **JP**  **ASH**  **FINAL** | **Unclear**  **Yes**  **Yes** |
| Question 3 | **JP**  **ASH**  **FINAL** | **Yes**  **N/A**  **N/A** |
| Question 4 | **JP**  **ASH**  **FINAL** | **No**  **No**  **No** |
| Question 5 | **JP**  **ASH**  **FINAL** | **N/A**  **No**  **N/A** |
| Question 6 | **JP**  **ASH**  **FINAL** | **Yes**  **Yes**  **Yes** |
| Question 7 | **JP**  **ASH**  **FINAL** | **Yes**${}^{\mathbf{1}}$**^,2,3,4^**  **Yes**${}^{\mathbf{1}}$**^,2,3,4^**  **Yes**${}^{\mathbf{1}}$**^,2,3,4^** |
| Question 8 | **JP**  **ASH**  **FINAL** | **Yes**${}^{\mathbf{1}}$**^,2,3,4^**  **Yes**${}^{\mathbf{1}}$**^,2,3,4^**  **Yes**${}^{\mathbf{1}}$**^,2,3,4^** |
| Question 9 | **JP**  **ASH**  **FINAL** | **Unclear^1,3,4^/Yes** ${}^{\mathbf{2}}$  **Unclear^3,4^/Yes** ${}^{\mathbf{1,2}}$  **Unclear^3,4^/Yes** ${}^{\mathbf{1,2}}$ |
| Question 10 | **JP**  **ASH**  **FINAL** | **Yes**${}^{\mathbf{1}}$**^,2,3,4^**  **Yes**${}^{\mathbf{1}}$**^,2,3,4^**  **Yes**${}^{\mathbf{1}}$**^,2,3,4^** |
| Question 11 | **JP**  **ASH**  **FINAL** | **Yes ^1,4^/Unclear** ${}^{\mathbf{2,3}}$  **Yes ^1,2,,3,4^**  **Yes ^1,2,,3,4^** |
| Question 12 | **JP**  **ASH**  **FINAL** | **Yes**${}^{\mathbf{1}}$**^,2,3,4^**  **Yes**${}^{\mathbf{1}}$**^,2,3,4^**  **Yes**${}^{\mathbf{1}}$**^,2,3,4^** |
| Question 13 | **JP**  **ASH**  **FINAL** | **Yes**  **Yes**  **Yes** |

1: accuracy of patient generated diagnosis assessed by matching >= 2/3 diagnoses with the clinician 2: anxiety 3: patient-clinician relationship 4: satisfaction
